# Investigating the psychophysiological effects of NaiKan Therapy: salivary oxytocin and cortisol release

**DOI:** 10.3389/fnint.2025.1476654

**Published:** 2025-02-25

**Authors:** Ming Qian, Minghui Wang, Siyi Song, Hansong Xia, Rui Huang, Qin Yuan, Zhi Zhu, Haiyan Wei, Ming Chen, Qing Ma, Hui Zhang

**Affiliations:** ^1^Nanhui Mental Health Center, Shanghai, China; ^2^Fudan University, Shanghai, China; ^3^Shanghai Sipo Polytechnic, Shanghai, China

**Keywords:** NaiKan Therapy, salivary oxytocin, salivary cortisol, psychophysiology, stress resilience

## Abstract

NaiKan Therapy, a method of self-reflection and introspection, has garnered considerable interest for its psychological benefits. However, its physiological impacts, particularly on hormonal regulation, remain underexplored. In this study, we aimed to investigate the effects of NaiKan Therapy on salivary oxytocin and cortisol release, shedding light on the psychophysiological mechanisms underlying this introspective practice. Sixty participants underwent Naikan Therapy sessions over five consecutive days, during which salivary samples were collected at multiple time points. Salivary oxytocin and cortisol levels were measured using enzyme-linked immunosorbent assay (ELISA) kits. Our results revealed significant increases in salivary oxytocin levels following NaiKan Therapy, suggesting a potential role of this practice in enhancing social bonding and emotional regulation. Conversely, salivary cortisol levels exhibited a decrease post-therapy, indicating a reduction in stress reactivity. These findings provide novel insights into the neuroendocrine mechanisms underlying NaiKan Therapy and highlight its potential as a holistic approach to improving mental wellbeing. Further research exploring the long-term effects of NaiKan Therapy and its implications for clinical practice is warranted.

## Introduction

NaiKan Therapy, a traditional Japanese method rooted in mindfulness and Naikan practices, has gained attention for its potential therapeutic benefits in promoting mental wellbeing and stress reduction (Ozawa-de Silva, 2015). Central to NaiKan Therapy is the practice of inward reflection, where individuals engage in deep introspection to explore their inner thoughts, emotions, and experiences. While anecdotal evidence suggests its efficacy in enhancing emotional resilience and fostering self-awareness, the underlying psychophysiological mechanisms of NaiKan Therapy remain largely unexplored (Ozawa-de Silva, 2015; Samuel, 2016). While NaiKan Therapy is inherently subjective and often difficult to measure objectively, recent advances in psychobiological research have shed light on its potential physiological correlates, including alterations in hormonal signaling.

Two key hormones that have garnered significant attention in the context of social cognition, emotional processing, and stress regulation are oxytocin and cortisol. Oxytocin, known for its role in social bonding, trust, and affiliative behaviors, is implicated in a wide range of social interactions and emotional experiences (Ross and Young, 2009; Olff et al., 2013). Cortisol, the primary glucocorticoid in rodents, serves as a crucial mediator of the stress response and plays a pivotal role in regulating various physiological and behavioral responses to stressors (Nicolaides et al., 2014; Gray et al., 2017).

While previous studies have demonstrated the influence of external social cues and stressors on oxytocin and cortisol release (Olff et al., 2013; Smith and Wang, 2012), relatively little is known about the effects of NaiKan Therapy on these hormonal systems. Given the intimate connection between NaiKan Therapy and self-awareness, as well as the potential emotional and cognitive implications of self-reflection (Machizawa and Enns, 2015), investigating the hormonal responses associated with NaiKan Therapy represents a novel and intriguing avenue of inquiry.

Salivary measurements offer a non-invasive and convenient method for assessing hormonal changes in response to psychological stimuli (Groschl, 2008; Clow and Smyth, 2020; Giacomello et al., 2020). Recent advances in salivary bioscience have enhanced the sensitivity and specificity of oxytocin and cortisol assays, allowing for more accurate detection of subtle hormonal fluctuations associated with psychological states (İnanıcı et al., 2024; Schneider et al., 2023; Moscovice et al., 2022; Bowling et al., 2022). Studies have demonstrated that examinations, a common academic stressor, lead to heightened salivary cortisol secretion, emphasizing the hormone’s role in stress responses (İnanıcı et al., 2024; Špiljak et al., 2022). In addition, this methodology can be utilized to assess the potential application value of stress management interventions (Špiljak et al., 2024; Mizuhata, 2021). These recent literatures suggest that mindfulness-based interventions and introspective therapies may modulate stress-related hormonal pathways, highlighting the need to explore the specific role of oxytocin and cortisol in NaiKan Therapy. By examining how NaiKan Therapy activities, such as self-reflection, self-awareness, and metacognition, influence salivary hormone levels, we can gain valuable insights into the psychophysiological mechanisms underlying NaiKan Therapy processes.

In this research paper, we aim to investigate the psychophysiological effects of NaiKan Therapy by examining its influence on salivary oxytocin and cortisol levels. We hypothesize that engaging in NaiKan Therapy will lead to alterations in salivary hormone levels, indicative of changes in emotional state and stress response. Through a series of controlled experiments involving participants undergoing NaiKan Therapy sessions, we will assess salivary oxytocin and cortisol levels before and after the intervention. Additionally, we will explore potential moderators, such as individual differences in trait mindfulness and baseline stress levels, to better understand the variability in hormonal responses to NaiKan Therapy.

## Materials and methods

### Participants

Participants, aged between 18 and 45 years old, were recruited for the study, totaling 60 individuals, both males and females (Table 1). Participants who dropped out before completing the intervention or had incomplete saliva samples were excluded from the final analysis. The sample size was determined based on practical considerations. Given the exploratory nature of this study and resource constraints, a sample size of 60 was deemed sufficient to detect meaningful hormonal changes while ensuring feasibility. Prior to the experiments, participants were provided with detailed verbal and written information regarding the study procedures and gave their informed consent. All experimental procedures were approved by the Shanghai Nanhui Mental Health Center Ethics Committee (No. 2023-C-002-E02).

**Table 1:** General information about the participants.

	Female	Male	*p*-value
Number of participants	37	23	
Age (years)	25.2 ± 1.8	20.3 ± 0.9	0.0479^1^
Previous COVID-19 infection	31/37	17/23	0.5081^2^
Hypertension	–	–	–
Diabetes	–	2/23	0.1429^2^
Coronary heart disease	–	–	–
Chronic obstructive pulmonary disease	–	–	–
Tumor	–	–	–
Stroke	–	–	–

1 Unpaired t test.

2 Fisher’s exact test.

### NaiKan Therapy

In intensive NaiKan Therapy (INT), the participant sits in the corner of a room, walled off by a folding screen to cut off visual stimulation from the outside so that it is easier for them to observe their own thinking. Sitting in a quiet place and staying in a relaxed position, the participant begins to seriously look into his/her thoughts, continuing his/her introspection daily from 06.00 h to 08.00 h (five consecutive days). The participants examine how they have lived according to three themes: (i) What have I received from a particular person? (ii) What have I returned to that person? and (iii) What troubles and difficulties have I caused that person? To begin with, the participants are asked to examine the relationship with their mothers or their main caretakers through every period of their life, starting from childhood and gradually moving to the present. Then, they are asked to examine themselves regarding other people who are close to them, such as their fathers, spouses, friends, colleagues, and so forth (Sengoku et al., 2010).

### Assessment of anxiety

Assessment of anxiety levels was conducted using the PHQ-9 (Patient Health Questionnaire-9) and DASS (Depression Anxiety Stress Scales) (Peters et al., 2021; Sun et al., 2020) before and after the NaiKan Therapy. These scales are widely utilized to measure individuals’ levels of depression and anxiety by inquiring about their psychological and emotional states over the past week. The PHQ-9 covers a range of depressive symptoms such as mood, insomnia, and fatigue, while the DASS provides a comprehensive evaluation of depression, anxiety, and stress. Through these assessment tools, changes in participants’ anxiety levels before and after the introspection sessions were quantified, allowing for an evaluation of the effects of NaiKan Therapy on anxiety symptoms. The cutoff score was set 50.

### Saliva sample collection

**Figure 1:**
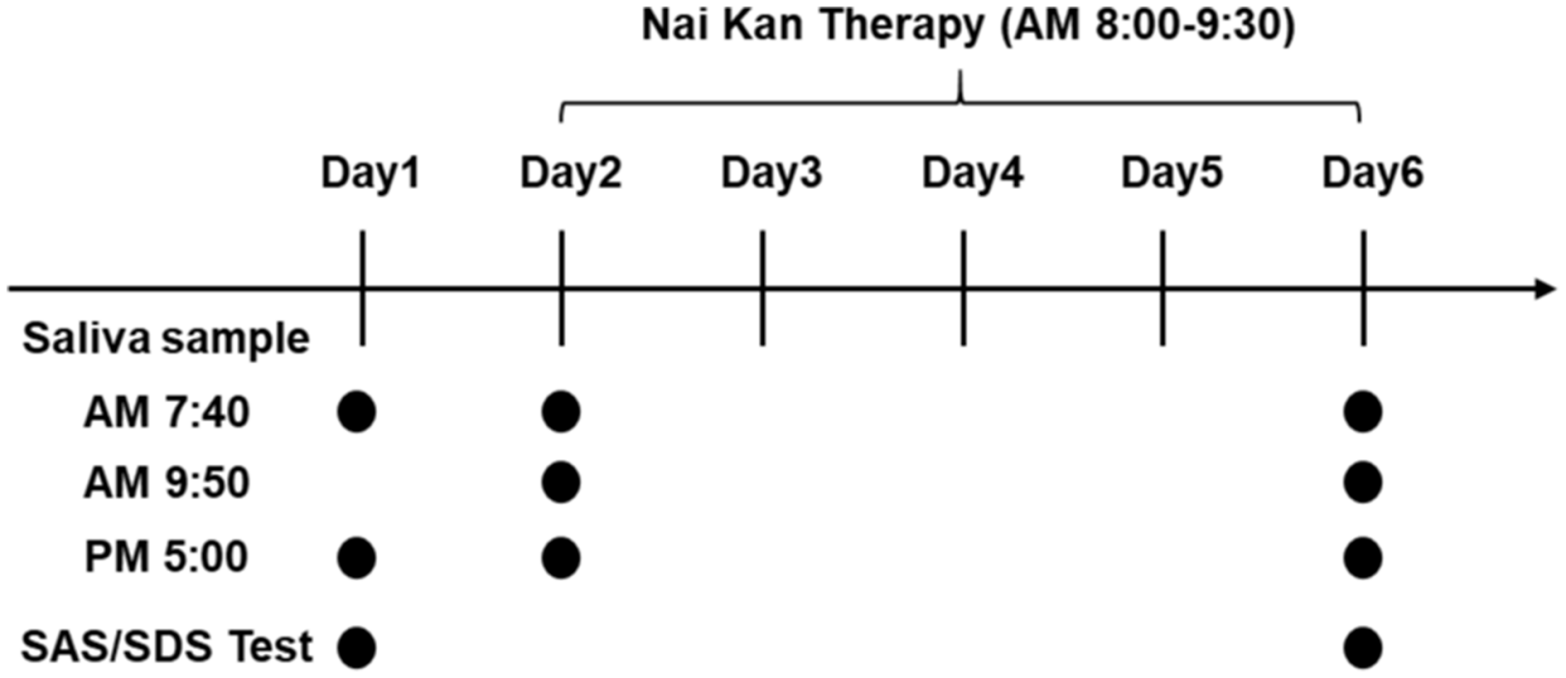
Experimental timeline.

Saliva samples were collected from participants following standardized protocols (Bellagambi et al., 2020; Szabo and Slavish, 2021). Participants were instructed to abstain from eating, drinking (except water), brushing teeth, using mouthwash, chewing gum, or undergoing dental procedures for at least 60 min prior to sample collection. Saliva samples were collected at different time points: upon waking (between 6:00 and 8:00 AM), 20 min before and after the Nai Kan Therapy session, and 30 min before dinner (between 5:00 and 7:00 PM) (Figure 1, Day 1, Day 2 and Day 6). Upon collection, saliva samples were centrifuged to obtain clear saliva supernatant. The supernatant was then transferred to new tubes for subsequent analysis.

### Enzyme-linked immunosorbent assay (ELISA)

Saliva samples were analyzed for oxytocin and cortisol levels using commercially available ELISA kits (D751010 for oxytocin, D711340 for cortisol, Songon, China) (López-Arjona et al., 2024). Quantification of oxytocin concentrations was commercially performed by radioimmunoassay with a sensitivity of 0.1–0.5 pg/sample as described previously (Schladt et al., 2017). Intra- and inter-assay coefficients for oxytocin were <10 and <12%, respectively. Quantification of cortisol concentrations was performed using a commercially available chemiluminescence immunoassay with high sensitivity. Intra- and inter-assay coefficients for cortisol were below 8% (Schladt et al., 2017). ELISA plates were coated with specific capture antibodies for oxytocin or cortisol and incubated to allow antibody binding. Standard solutions with known concentrations of oxytocin or cortisol were prepared, and serial dilutions of these standards were added to the ELISA plates to generate standard curves.

Saliva samples and standards were added to appropriate wells of the ELISA plate and incubated to allow oxytocin or cortisol in the samples to bind to the capture antibodies. After washing to remove unbound substances, detection antibodies specific to oxytocin or cortisol were added, followed by a secondary antibody conjugated to an enzyme. Substrate solution was then added to initiate an enzymatic reaction, resulting in a color change.

The absorbance of each well was measured at a specific wavelength using a microplate reader, and absorbance values were recorded for subsequent analysis. Standard curves were generated using the absorbance values of the standards, and the concentrations of oxytocin and cortisol in the saliva samples were interpolated from these curves. Statistical analysis was performed to analyze the data, including calculation of means, standard deviations, and statistical significance where applicable. Quality control measures were implemented throughout the assay to ensure accuracy and reliability of the results.

### Statistics

All data were analyzed by GraphPad 8.0 with *p* ≤ 0.05 considered statistically significant. All data are shown as means ± S.E.M. Changes in salivary oxytocin and cortisol were analyzed using one-way ANOVA for repeated measures. Since salivary oxytocin and cortisol concentrations were not normally distributed and basal concentrations showed considerable individual variability, these values were normalized to percentage of their corresponding baseline as follows: (value *x*)/(value of Basal)*100% (Schladt et al., 2017). Multiple comparisons were adjusted using Bonferroni correction or false discovery rate correction. To ensure the stability and reliability of the statistical analysis, only participants with complete data were included. Participants who withdrew before completing the intervention or had incomplete saliva samples were excluded from the final statistical analysis. Given the exclusion of incomplete data, no data imputation methods were necessary. Subgroup analyses investigated differences based on participant characteristics. Results were reported descriptively with effect sizes, *p*-values, and presented in figures legends.

## Results

### Improvement in anxiety and depression symptoms with NaiKan Therapy

**Figure 2:**
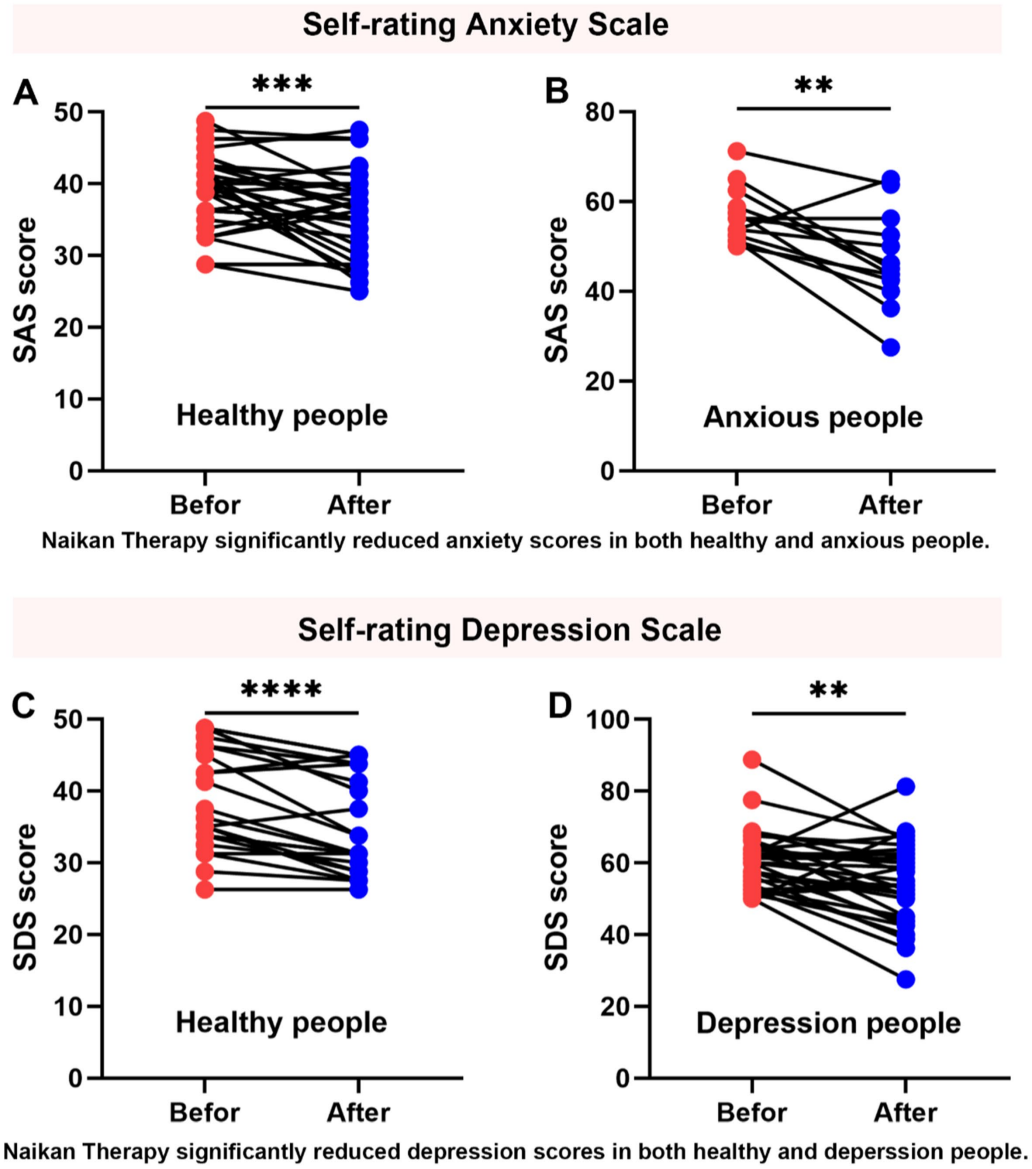
Improvement in anxiety and depression symptoms with NaiKan Therapy. **(A)** Anxiety scores of normal subjects before and after training (before: 39.04 ± 0.88, after: 35.79 ± 1.01, *n* = 35, paired t test, *p* = 0.0007). **(B)** Anxiety scores of anxious subjects before and after training (before: 56.92 ± 1.71, after: 47.12 ± 2.91, *n* = 13, paired t test, *p* = 0.0036). **(C)** Depression scores of normal subjects before and after training (before: 39.01 ± 1.47, after: 35.21 ± 1.38, *n* = 24, paired t test, *p* < 0.0001). **(D)** Depression scores of depression subjects before and after training (before: 60.11 ± 1.42, after: 54.67 ± 1.90, *n* = 34, paired t test, *p* = 0.0037). The values presented are raw measurements. Means ± SEMs.

Prior to engaging in introspection sessions, the cohort of 60 individuals underwent assessment using the Zung Self-Rating Anxiety Scale (SAS) and Zung Self-Rating Depression Scale (SDS). Based on these evaluations, 26 participants were categorized as having no symptoms of anxiety or depression, while 34 individuals exhibited symptoms indicative of anxiety and depression. Following a structured regimen of NaiKan Therapy spanning 5 days, significant improvements were observed in SAS and SDS scores across both groups (Figure 2).

To evaluate the effects of training on anxiety and depression, we conducted paired t-tests comparing pre- and post-training scores in both normal and affected participants. For anxiety, normal subjects exhibited a significant reduction in scores following training (before: 39.04 ± 0.88, after: 35.79 ± 1.01, *n* = 35, paired t-test, *p* = 0.0007). Similarly, anxious subjects showed a significant decrease in anxiety scores post-training (before: 56.92 ± 1.71, after: 47.12 ± 2.91, *n* = 13, paired t-test, *p* = 0.0036) (Figures 2A,B). For depression, normal subjects also demonstrated a significant reduction in scores after training (before: 39.01 ± 1.47, after: 35.21 ± 1.38, *n* = 24, paired t-test, *p* < 0.0001). Additionally, individuals with depression exhibited a significant decrease in depression scores following training (before: 60.11 ± 1.42, after: 54.67 ± 1.90, *n* = 34, paired t-test, *p* = 0.0037) (Figures 2C,D). These results suggest that NaiKan Therapy holds potential as an effective intervention for alleviating symptoms of anxiety and depression, warranting further investigation into its therapeutic mechanisms and long-term efficacy.

### Effect of NaiKan Therapy on salivary oxytocin and cortisol

**Figure 3:**
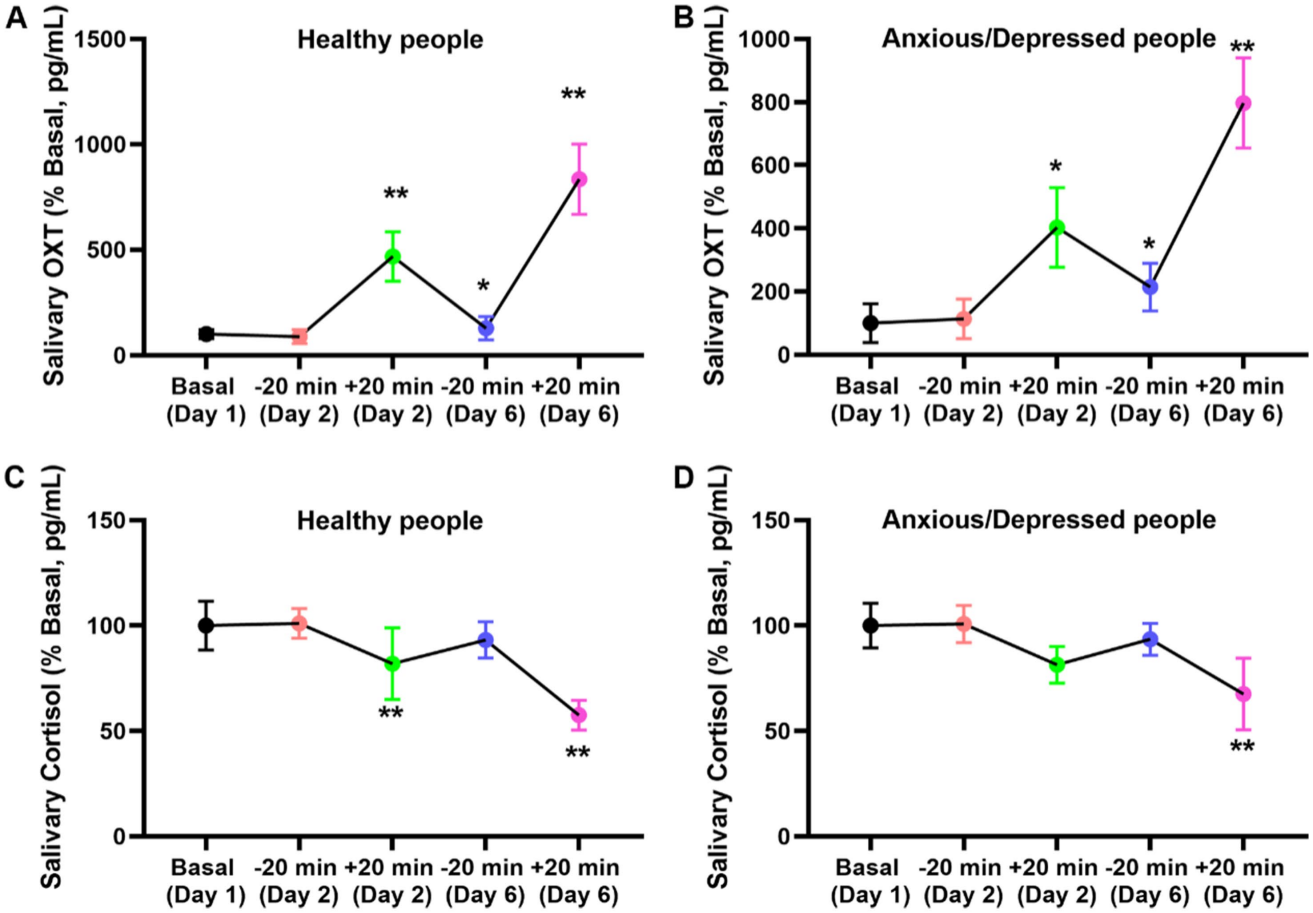
Effect of NaiKan Therapy on salivary oxytocin and cortisol. **(A)** Oxytocin concentration of normal subjects before and after training (Basaline [Day 1]: 100.00 ± 21.24, −20 min [Day 2]: 87.78 ± 32.23, +20 min [Day 2]: 467.59 ± 117.40, −20 min [Day 6]: 128.42 ± 55.30, +20 min [Day 6]: 834.59 ± 166.12, *n* = 26, One-way ANOVA, *F*_(4, 125)_ = 11.59, *p* < 0.0001). **(B)** Oxytocin concentration of anxious/depression subjects before and after training (Basaline [Day 1]: 100.00 ± 61.24, −20 min [Day 2]: 113.72 ± 62.60, +20 min [Day 2]: 402.91 ± 125.50, −20 min [Day 6]: 214.53 ± 75.40, +20 min [Day 5]: 797.21 ± 143.41, *n* = 34, One-way ANOVA, *F*_(4, 165)_ = 8.47, *p* < 0.0001). **(C)** Cortlsol concentration of normal subjects before and after training (Basaline [Day 1]: 100.00 ± 11.51, −20 min [Day 2]: 101.09 ± 7.01, +20 min [Day 2]: 81.98 ± 17.05, −20 min [Day 6]: 93.24 ± 8.60, +20 min [Day 6]: 57.50 ± 7.11, *n* = 26, One-way ANOVA, *F*_(4, 125)_ = 2.735, *p* = 0.0319). **(D)** Cortlsol concentration of anxious/depression people before and after training (Basaline [Day 1]: 100.00 ± 10.57, −20 min [Day 2]: 100.79 ± 8.81, +20 min [Day 2]: 81.38 ± 8.65, −20 min [Day 6]: 93.54 ± 7.56, +20 min [Day 6]: 67.55 ± 17.02, *n* = 34, One-way ANOVA, *F*_(4, 165)_ = 2.634, *p* = 0.0381). The values presented are baseline-adjusted. Means ± SEMs.

Our investigation into the impact of introspection therapy on salivary oxytocin and cortisol levels revealed noteworthy trends. Initially, individuals with anxiety and depression exhibited elevated baseline oxytocin and cortisol levels compared to their healthy counterparts. However, following introspection sessions, oxytocin and cortisol levels decreased uniformly within 20 min, irrespective of the participants’ initial status. Importantly, prolonged exposure to introspection over 5 days resulted in a change in baseline oxytocin and cortisol levels among individuals with anxiety and depression (Figure 3).

For oxytocin levels in normal subjects, a significant increase was observed after training (*F*_(4, 125)_ = 11.59, *p* < 0.0001). Baseline oxytocin concentration (Day 1) was 100.00 ± 21.24 pg/mL, with no significant change at −20 min on Day 2 (87.78 ± 32.23 pg/mL). However, a marked increase was detected at +20 min on Day 2 (467.59 ± 117.40 pg/mL). On Day 6, oxytocin concentration at −20 min was 128.42 ± 55.30 pg/mL, rising significantly to 834.59 ± 166.12 pg/mL at +20 min (Figure 3A). A similar trend was observed in anxious and depressed participants, with a significant overall effect of training on oxytocin levels (*F*_(4, 165)_ = 8.47, *p* < 0.0001). Baseline levels (Day 1) were 100.00 ± 61.24 pg/mL, with a slight increase at −20 min on Day 2 (113.72 ± 62.60 pg/mL). Notably, oxytocin levels significantly rose at +20 min on Day 2 (402.91 ± 125.50 pg/mL) and continued increasing at +20 min on Day 6 (797.21 ± 143.41 pg/mL) compared to their respective baseline values (Figure 3B). For cortisol levels, a significant reduction following training was observed in both normal (*F*_(4, 125)_ = 2.735, *p* = 0.0319) and anxious/depressed subjects (*F*_(4, 165)_ = 2.634, *p* = 0.0381). In normal participants, baseline cortisol levels (Day 1) were 100.00 ± 11.51 pg/mL, with no notable change at −20 min on Day 2 (101.09 ± 7.01 pg/mL). However, a significant reduction was observed at +20 min on Day 2 (81.98 ± 17.05 pg/mL), which persisted on Day 6 (+20 min: 57.50 ± 7.11 pg/mL) (Figure 3C). Similarly, in anxious and depressed participants, baseline cortisol levels were 100.00 ± 10.57 pg/mL, with a slight increase at −20 min on Day 2 (100.79 ± 8.81 pg/mL). However, a significant decline was observed at +20 min on Day 2 (81.38 ± 8.65 pg/mL), which was further reduced at +20 min on Day 6 (67.55 ± 17.02 pg/mL) (Figure 3D).

These findings suggest that introspection may exert an effect on oxytocin and cortisol secretion, potentially contributing to the amelioration of anxiety and depression symptoms over time.

### Correlation analysis of oxytocin and cortisol concentration changes with anxiety and depression ratings

To elucidate the relationship between hormonal changes and improvements in anxiety and depression symptoms, we conducted correlation analyses. Our results demonstrated a negative correlation between changes in oxytocin concentrations and anxiety and depression ratings, indicating that greater increases in oxytocin levels were associated with lower symptom severity. Conversely, changes in cortisol concentrations exhibited a positive correlation with anxiety and depression ratings, with greater reductions in cortisol levels corresponding to lower symptom severity. These findings underscore the intricate interplay between hormonal dynamics and psychological wellbeing following introspection therapy.

## Discussion

The results of our study align with prior research indicating the therapeutic potential of NaiKan Therapy in alleviating symptoms of anxiety and depression. Our findings corroborate those of previous studies that have reported significant improvements in mental health outcomes following Naikan interventions (Sengoku et al., 2010; Zhang et al., 2015). By demonstrating consistent reductions in both anxiety and depression scores, our study adds to the growing body of evidence supporting the efficacy of Naikan approaches in mental health management.

In addition to psychological assessments, the inclusion of saliva testing in our study provided several advantages for investigating the effects of Naikan therapy on mental health outcomes. Saliva sampling offers a non-invasive and convenient method for assessing neuroendocrine responses to psychological interventions (Giacomello et al., 2020). This non-invasive nature enhances participant compliance and reduces the burden associated with sample collection, facilitating longitudinal studies with multiple sampling time points. Moreover, saliva contains various biomarkers that reflect neuroendocrine activity, including oxytocin and cortisol, which play key roles in stress regulation and emotional processing (Young Kuchenbecker et al., 2021; Ali and Nater, 2020; Wirobski et al., 2024). By measuring these biomarkers, our study gained insights into the physiological mechanisms underlying the therapeutic effects of NaiKan Therapy. Some study indicates that conducting daily NaiKan Therapy is effective for maintaining the psychological and psychosomatic state at 3 months following the intensive NaiKan Therapy (Sengoku et al., 2010). Changes in salivary oxytocin and cortisol levels following Naikan interventions may indicate alterations in stress reactivity, emotion regulation, and social bonding processes (Olff et al., 2013; Young Kuchenbecker et al., 2021; Engert et al., 2016). Additionally, prior research suggests that oxytocin and cortisol in saliva are influenced by a variety of activities, such as exercise or music (Schladt et al., 2017; de Jong et al., 2015), raising the possibility that NaiKan Therapy may exert its effects through similar mechanisms. Therefore saliva testing allowed for a comprehensive assessment of the neurobiological pathways implicated in mental health disorders and their response to therapeutic interventions.

The differential response to NaiKan Therapy observed between individuals with and without symptoms of anxiety and depression is consistent with findings from studies investigating personalized treatment approaches (Yeshi, 2018; Captari et al., 2022). Tailoring interventions based on individual symptomatology and therapeutic response has emerged as a promising strategy for optimizing treatment outcomes and enhancing patient engagement (Rush and Thase, 2018; Ng and Weisz, 2016). Our results underscore the importance of considering individual differences in treatment planning and delivery to maximize the effectiveness of NaiKan Therapy.

The mechanisms underlying the therapeutic effects of NaiKan Therapy warrant further exploration, as highlighted by recent neuroscientific investigations. Neuroimaging studies have implicated neural circuits involved in self-awareness, emotion regulation, and cognitive control in mediating the effects of Naikan interventions (Herwig et al., 2010; Opialla et al., 2015). Additionally, biomarker analyses have revealed changes in neuroendocrine signaling pathways, including alterations in oxytocin and cortisol levels, following Naikan practices (Opialla et al., 2015; Iovino et al., 2021; Li et al., 2019). Oxytocin is primarily synthesized in the hypothalamus and plays a key role in social bonding, emotion regulation, and stress attenuation through its interactions with the amygdala and prefrontal cortex (Olff et al., 2013; Triana-Del Rio et al., 2022). Engaging in introspective practices such as NaiKan Therapy may stimulate oxytocin release by enhancing social cognition and fostering a sense of self-compassion and emotional regulation. Similarly, cortisol, a critical stress hormone regulated by the hypothalamic–pituitary–adrenal (HPA) axis, responds to psychological stressors by modulating energy metabolism, immune function, and emotional processing (Mbiydzenyuy and Qulu, 2024; Smith and Vale, 2006). Our findings suggest that NaiKan Therapy may mitigate HPA axis hyperactivity, thereby reducing cortisol levels and promoting stress resilience. However, it remains unclear whether these hormonal shifts are driven by Naikan-specific mechanisms or whether they arise from broader relaxation responses, placebo effects, or increased social bonding. Future research integrating neurobiological assessments with psychological measures could provide a comprehensive understanding of the underlying mechanisms of NaiKan Therapy.

A more comprehensive understanding of NaiKan Therapy’s effectiveness could be achieved by comparing it with other established psychological interventions, such as mindfulness-based stress reduction (MBSR) or cognitive behavioral therapy (CBT) (Fujisaki, 2020). Comparative studies could elucidate whether NaiKan Therapy offers unique benefits beyond general mindfulness practices. Furthermore, future investigations should explore how the observed hormonal changes might be leveraged in clinical settings to treat stress-related disorders. If NaiKan Therapy reliably modulates oxytocin and cortisol pathways, it could serve as an adjunct intervention for conditions such as generalized anxiety disorder, major depressive disorder, or post-traumatic stress disorder.

In conclusion, our study contributes to the growing body of evidence supporting the efficacy of NaiKan Therapy in improving mental health outcomes, particularly for individuals experiencing symptoms of anxiety and depression. By elucidating the mechanisms underlying its therapeutic effects and exploring its long-term sustainability, future research has the potential to enhance the accessibility and effectiveness of Naikan interventions in diverse clinical settings. Addressing the limitations of short-term follow-up, self-reported biases, and cultural generalizability will be crucial for advancing NaiKan Therapy as an evidence-based mental health intervention.

## Limitations

Despite the promising findings, several limitations should be acknowledged. First, our study evaluated only short-term hormonal changes following NaiKan Therapy, without incorporating long-term follow-ups. As a result, it remains uncertain whether the observed effects on oxytocin and cortisol are sustained or revert to baseline over time. Future research should include follow-up assessments (e.g., one week or one month post-therapy) to determine the durability of these effects. Longitudinal studies with extended follow-up periods are needed to assess the durability of treatment effects and identify factors contributing to sustained improvements in mental health outcomes (Breier et al., 1991; Winzer et al., 2018). Second, our reliance on self-reported anxiety and depression scores introduces a potential subjective bias. Although validated psychological scales were used, self-report measures remain susceptible to individual differences in perception and reporting. Future studies should incorporate clinician-administered assessments or physiological measures of stress reactivity to enhance the robustness of findings. Third, we did not control for external factors that may have influenced hormonal changes, such as participants’ engagement in other stress-reducing activities (e.g., meditation, exercise, or social interactions). These confounding variables could have contributed to the observed effects and should be addressed in future research through stricter control measures or study designs that directly compare NaiKan Therapy with other mindfulness-based interventions. Fourth, the sample size, while adequate for initial insights, may limit the generalizability of our results, particularly as the study primarily focused on individuals already exhibiting symptoms of anxiety and depression. Additionally, given that NaiKan Therapy has its origins in Japan, cultural influences may affect its generalizability to populations outside of East Asia (Shantha, 2019; Kirmayer, 2015). The introspective nature of NaiKan Therapy may resonate differently with individuals from Western cultures, where self-reflection is often conceptualized through different frameworks (Kirmayer, 2015). Future cross-cultural studies are needed to determine whether NaiKan Therapy’s effects extend across diverse populations.

## Data Availability

The original contributions presented in the study are included in the article/Supplementary material, further inquiries can be directed to the corresponding authors.
