# Supplementary material for: Investigating the psychophysiological effects of NaiKan Therapy: salivary oxytocin and cortisol release
Source: Front Integr Neurosci. 2025 Feb 25;19:1476654. doi: 10.3389/fnint.2025.1476654 (PMC11893859; doi:10.3389/fnint.2025.1476654)

**Investigating the Psychophysiological Effects of NaiKan Therapy: Salivary Oxytocin and Cortisol Release**

**Author:**

Ming Qian^1, #^, Minghui Wang^1^, Siyi Song^1^, Hansong Xia^1^, Rui Huang^1^, Qin Yuan^1^, Zhi Zhu^1^, Haiyan Wei^1^, Ming Chen^3^ Qing Ma^2^, Hui Zhang^2,^ ^#^.

^1^ Nanhui mental health center, Shanghai Pudong New Area, Shanghai China 201399

^2^ Shanghai Sipo Polytechnic, Shanghai China 201399

^3^ Fudan University

^#^Correspondence author: Ming Qian, [pdnjqm@163.com](mailto:pdnjqm@163.com); Hui Zhang, [18019256933@163.com](mailto:18019256933@163.com).


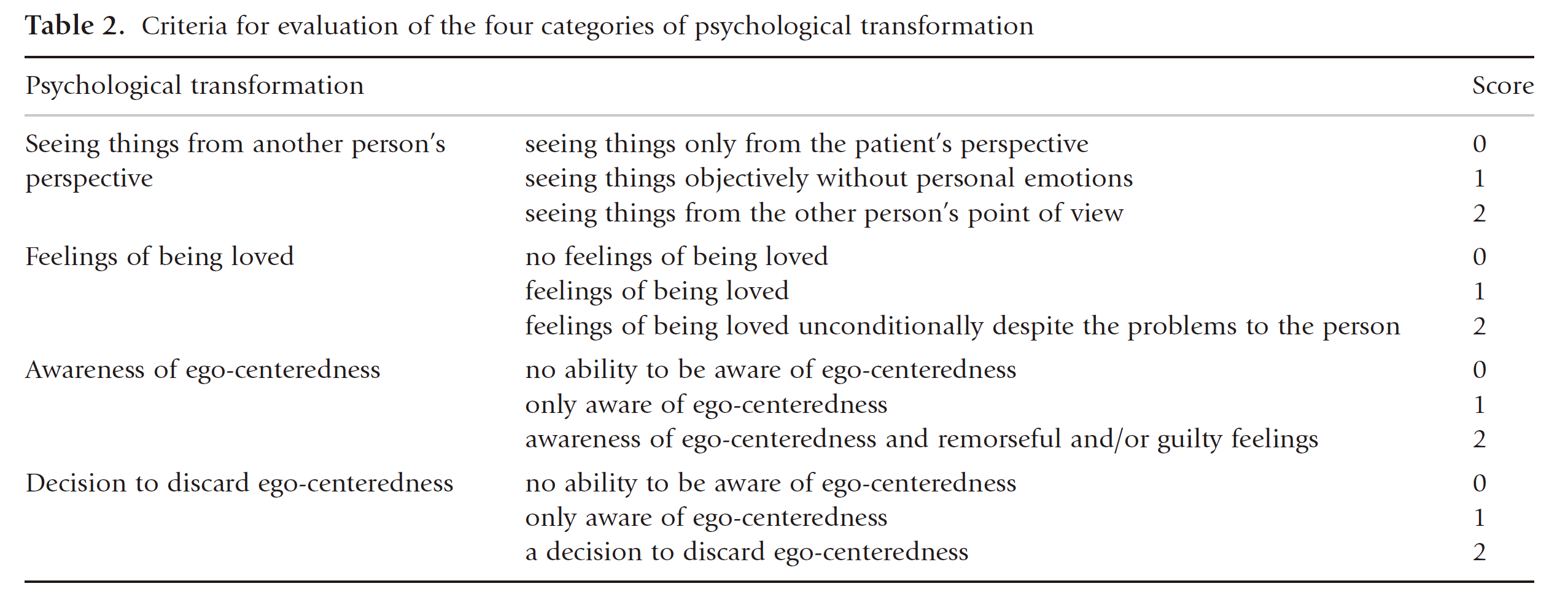


Table 1 Criteria for evaluation of four categories of psychological transformation in Naikan

Standard curve of oxytocin concentration


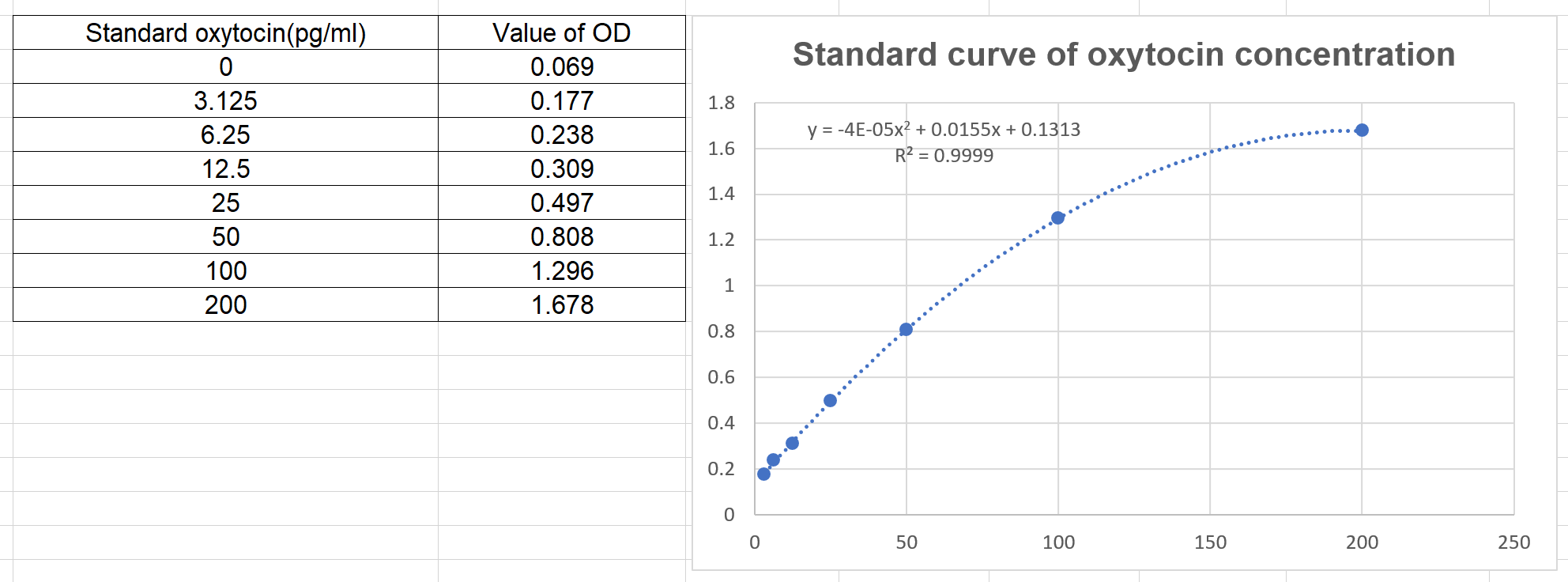


Standard curve of cortisol concentration


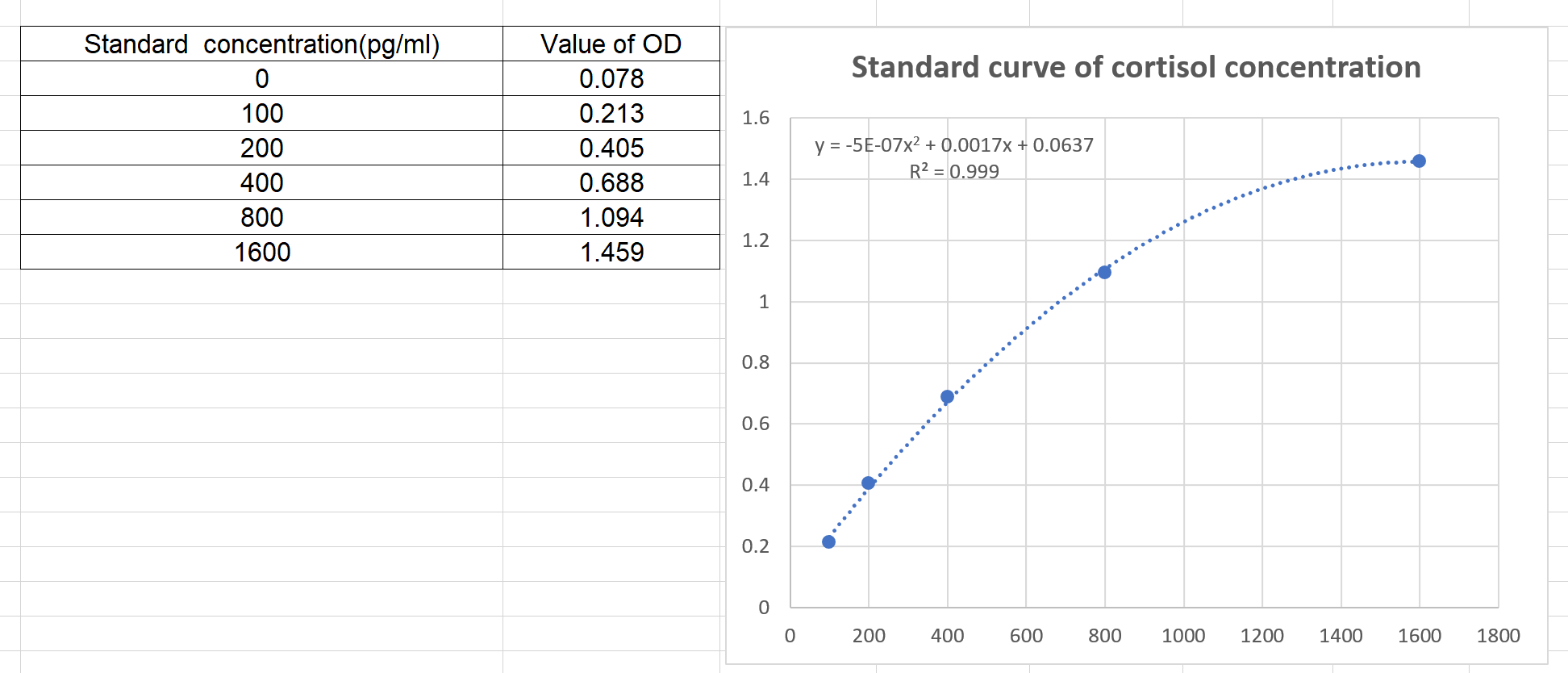

Supplement: Supplementary file 1 [file Table_1.docx]
